# Supplementary material for: The Cooperative Anti-Neoplastic Activity of Polyphenolic Phytochemicals on Human T-Cell Acute Lymphoblastic Leukemia Cell Line MOLT-4 In Vitro
Source: Int J Mol Sci. 2022 Apr 26;23(9):4753. doi: 10.3390/ijms23094753 (PMC9099961; doi:10.3390/ijms23094753)
Supplement: Supplementary file 1 [file ijms-23-04753-s001.zip › ijms-1671918-supplementary.pdf]

International Journal of *Molecular Sciences*

*Article*

# The cooperative anti-neoplastic activity of polyphenolic phytochemicals on human T-cell acute lymphoblastic leukemia cell line MOLT-4 *in vitro*

**Patrycja Koszałka [1,†], Grzegorz Stasiłojć [1,†], Natalia Miękus-Purwin [2], Maciej Niedźwiecki [3], Maciej Purwin [2], Szymon Grabowski [4], Tomasz Bączek [2,*]**

## Supplemental Material

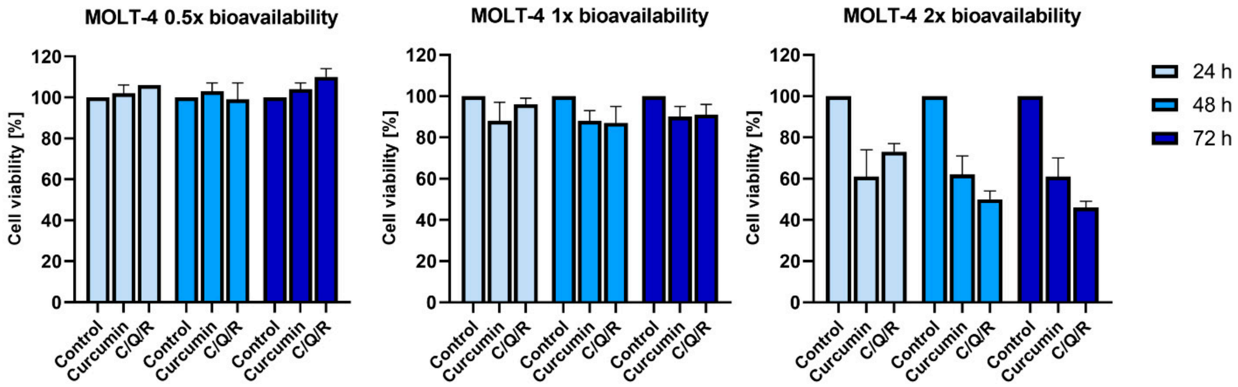


**Figure S1.** Comparison in a percentage of viable MOLT-4 leukemia cells treated with curcumin and its combination with quercetin and resveratrol without genistein. Nutraceuticals were used at a comparable dose according to their bioavailability. Bioavailable concentration specific for each dietary polyphenol at 0.5x / 1x / 2x dose: curcumin 3.05 μM / 6.1 μM / 12.2 μM, genistein 5.55 μM / 11.1 μM / 22.2 μM, quercetin 2.48 μM / 4.96 μM / 9.92 μM, or resveratrol 1.1 μM / 2.2 μM / 4.4 μM, respectively. Viability was measured with MTT metabolic assay after 24, 48, and 72 h with non-treated control (C) representing 100% viability. All data points represent averages of three independent tests. Standard deviations are marked.

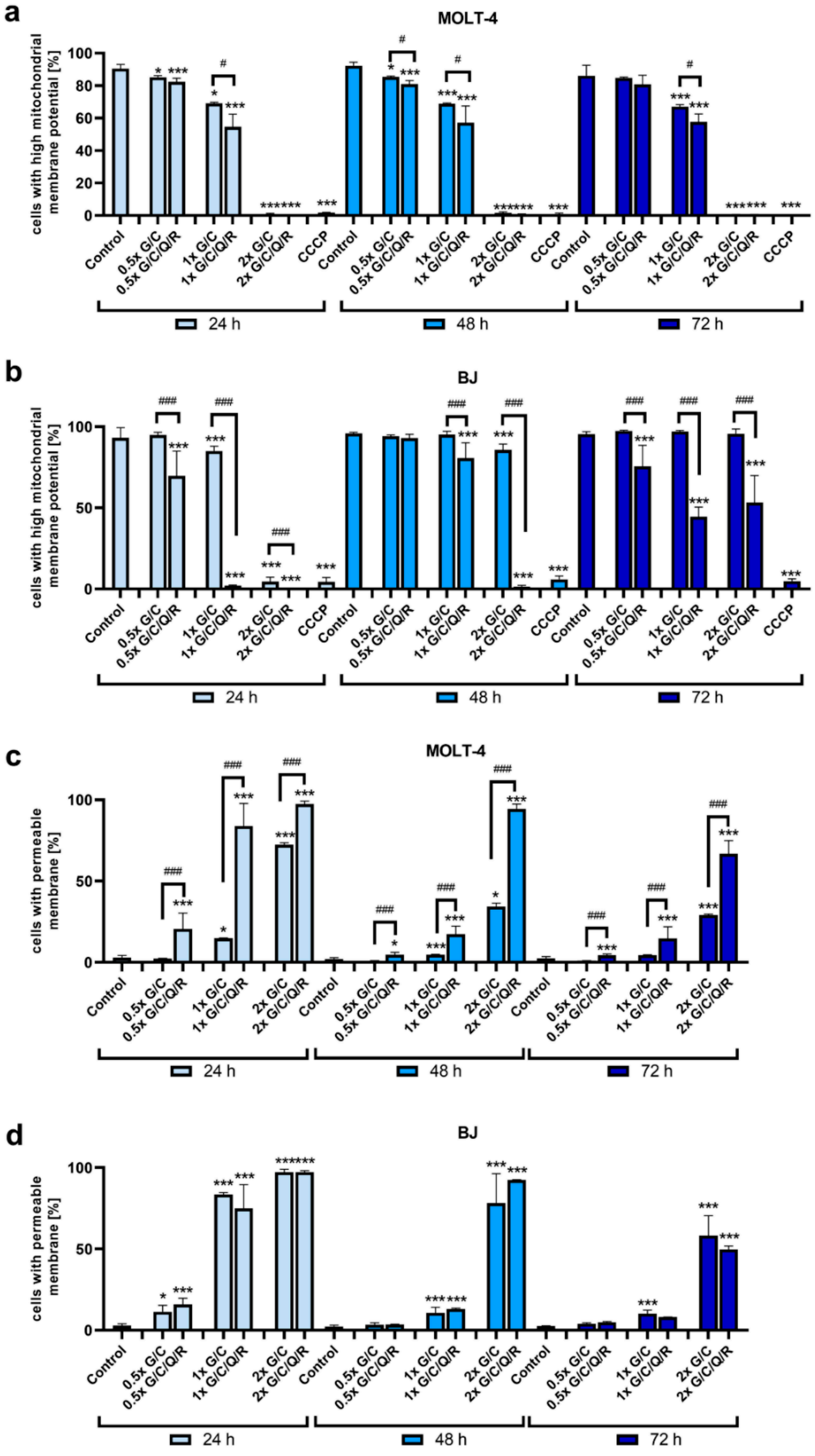


**Figure S2.** Changes in mitochondrial membrane potential and cell membrane permeability induced by a combination of nutraceuticals. Cells were treated for 24, 48, and 72 h with a mix of genistein and curcumin (G/C) or genistein/curcumin/quercetin/resveratrol (G/C/Q/R) combined at a comparable dose according to their bioavailable concentration at 0.5x / 1x / 2x dose: curcumin 3.05 μM / 6.1 μM / 12.2 μM, genistein 5.55 μM / 11.1 μM / 22.2 μM, quercetin 2.48 μM / 4.96 μM / 9.92 μM, or resveratrol 1.1 μM / 2.2 μM / 4.4 μM, respectively. **a-b** Mitochondrial membrane potential (MMP) of MOLT-4 (**a**) and BJ cells (**b**) was measured with non-treated cells as a negative control, and CCCP-treated cells as a positive control for low MMP. **c-d** Cell membrane permeability (CMP) of MOLT-4 (**c**) and BJ cells (**d**) was measured with non-treated cells as a negative control. The data are presented as the mean ± SD of three independent tests (*P<0.05, ***P<0.001 vs. respective control).

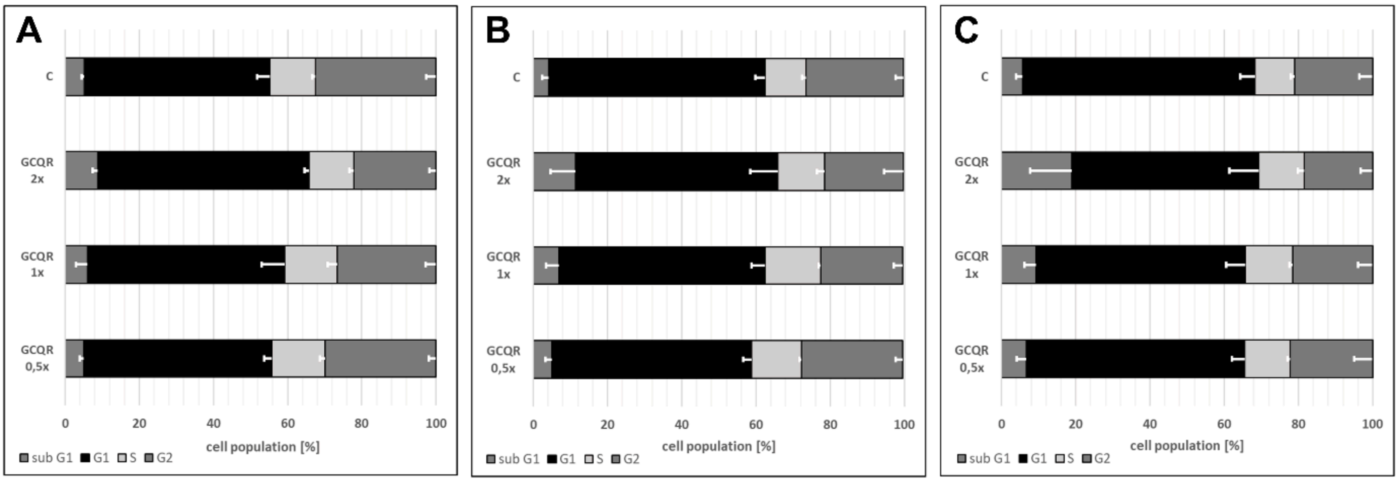


**Figure S3.** Percentage of MOLT-4 cell population cells in cell cycle during cell culture and treatment with nutraceuticals. A) 24h B) 48h C) 72h. MOLT-4 cell line was treated for 24, 48 and 72 h with a comparable dose of nutraceuticals according to their bioavailability. Untreated cells were used as a control (C). All data points represent averages of three independent tests. There were no statistically significant differences between untreated and treated cells in all treatment times and for all analysed cell cycle stages.
